# Temporal stability and change in manifest intelligence scores: Four complementary analytic approaches

**DOI:** 10.1016/j.mex.2021.101613

**Published:** 2021-12-23

**Authors:** Moritz Breit, Vsevolod Scherrer, Franzis Preckel

**Affiliations:** Department of Psychology, University of Trier, Germany

**Keywords:** Stability, Change, Test-retest, Intelligence profiles, Continuity

## Abstract

The temporal stability of psychological test scores is one prerequisite for their practical usability. This is especially true for intelligence test scores. In educational contexts, high stakes decisions with long-term consequences, such as placement in special education programs, are often based on intelligence test results. There are four different types of temporal stability: mean-level change, individual-level change, differential continuity, and ipsative continuity. We present statistical methods for investigating each type of stability. Where necessary, the methods were adapted for the specific challenges posed by intelligence research (e.g., controlling for general intelligence in lower order test scores). We provide step-by-step guidance for the application of the statistical methods and apply them to a real data set of 114 gifted students tested twice with a test-retest interval of 6 months.

• Four different types of stability need to be investigated for a full picture of temporal stability in psychological research

• Selection and adaption of the methods for the use in intelligence research

• Complete protocol of the implementation

## Graphical abstract

**Figure:**
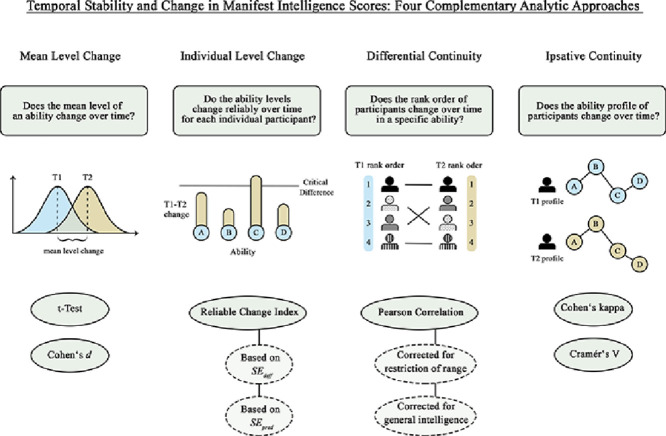

Specifications table

**Table:** 

Subject Area	Psychology
More specific subject area	*Intelligence research*
Method name	*Temporal stability*
Name and reference of original method	*t-Test* *Cohen's d* *Reliable change index* *Pearson correlation* *Profile reliability* *Cohen's kappa* *Cramér's V*
Resource availability	*Not applicable.*

## Introduction

Intervention decisions based on intelligence testing often have long-term consequences for individuals, such as admission to gifted classes or special education placement. Therefore, it is important to examine whether intelligence tests scores exhibit sufficient temporal stability. Whereas general intelligence has been consistently found to be a highly stable trait [13,23], the temporal stabilities of lower-order ability scores and ability profiles are more controversial (e.g., [6,37]) and require further research. In the present article, we describe four types of temporal stability, provide step-by-step guidance on how to assess these types of temporal stability, and apply the described methods to a sample of 114 students assessed twice over a 6-month interval. The methods can also be applied to test the temporal stability of other psychological constructs such as personality traits or motivational variables.

Importantly, we focus on the stability of manifest test scores, as these are frequently used in intelligence testing practice. The methods described in this paper are therefore ideally used to evaluate the stability of the scores of a specific test instrument to decide on its appropriateness for diagnostic decisions with long-term consequences or to replicate studies on the stability of lower level intelligence test scores (e.g., [22,37]). To evaluate the temporal stability of underlying cognitive abilities, latent variable approaches are more appropriate than the methods presented in the present article.

In previous studies investigating the temporal stability of intelligence, individual aspects of stability were investigated in isolation (e.g., [22,37]). We present a protocol for the combined investigation of all aspects for a full picture of temporal stability in hierarchically organized, multidimimensional psychological variables and intelligence in particular. Specific adaptions of the methods or the protocol for the use in intelligence test scores are marked with an asterix (*).

### Types of temporal stability description and step-by-step guidance

Four different types of temporal stability can be investigated in psychological research [12,29]. We present different statistical methods for the investigation of each type of stability. These methods are complementary and it often makes sense to test all four types to obtain a full picture of temporal stability. Nevertheless, some guidance regarding which method is especially appropriate for which application is presented at the end of this section. Many of these methods can easily be implemented using standard statistical software such as SPSS or R. When calculations have to be performed manually, we provide an illustrative example. R-code to replicate all analyses can be found in the Appendix.

*1. Mean-level change.* The mean-level change represents the change in the mean value of a variable across time within a sample. Investigations of mean-level change answer the question whether the score increases, decreases, or remains stable over time. As IQ-scores are standardized within age groups with a constant mean of 100, no major mean-level change would usually be expected beyond common retest effects, which have been quantified in a meta-analysis [30]. If the observed mean-level change far exceeds the expected retest effect (i.e., gain of 4.5 IQ-points in a year), this may indicate that the test allows for better item memorization or more advantage from general test familiarity than other cognitive tests. It may also indicate that some event or intervention between tests improved cognitive ability.

In our real data example, we tested whether our sample of students on average exhibited relevant increases in general intelligence and in more specific intelligence scores after six months. The statistical significance of the mean-level change can be tested by paired-samples *t*-test.

(1) $$t=\frac{{\bar{X}}_{D}}{s_{D}/\sqrt{n}}$$

In this formula, ${\bar{X}}_{D}$is the average difference between T1 and T1, $s_{D}$ is the standard deviation of this difference, and *n* is the sample size. A *p* value < .05 indicates that the likelihood that the observed average differences between the means at T1 and T2 occurred by chance is smaller than 5%. However, a significant paired-samples *t*-test does not quantify the size of the effect. Cohen's *d* is an effect size that can be used to quantify the magnitude of the observed change and to indicate whether the mean-level change was non-relevant (<.2), small (.2–.49), medium-sized (.5–.79), or large (>.8). It standardizes the difference between T1 and T2 on the first standard deviation.

(2) $$d=\frac{M_{t2}-M_{t1}}{SD_{t1}}$$

Cohen's *d* is calculated by subtracting the T1 sample mean (*M_t1_*) from the T2 sample mean (*M_t2_*) and by dividing the resulting difference by the standard deviation at T1. In the literature, this effect size is also labeled as pretest-posttest raw score effect size or Glass's Δ [24,31]. Note that in the original Cohen's *d* formula, the mean difference is divided by the pooled standard deviation of T1 and T2 [9]. However, Becker [1] argued that posttest standard deviation could be influenced by the previous testing, whereas the standard deviation at T1 is free of either influence. According to Cohen [9], *d* = .20 represents a small difference, *d* = .50 represents a medium-size difference, and *d* = .80 represents a large difference between mean values. That is, if IQ scores were assessed twice and the T1 sample mean was 100 (*SD_t1_* = 15) and the T2 sample mean was 110 (*SD_t2_* = 13), a medium sized increase (*d* = .67) occurred over time.

*2. Individual-level change.* The individual-level change represents the reliability of change in individuals. The reliable change index (RCI) [16] reveals the extent to which observed individual changes in a score occurred due to measurement error or due to meaningful change. Thus, it can be investigated whether the individual participants showed statistically significant increases, decreases, or no change in the investigated variable over time. In intelligence testing, one would usually not expect a large number of participants with significant individual-level change of their test scores, especially within short test-retest intervals. A large proportion of significant individual-level changes in the same direction may therefore point towards differences in the test situation between first and second testing or to item memorization and test familiarity effects. Alternatively, individual-level change may be used to investigate interindividual differences in the effect of a specific intervention on intelligence test scores. Here, one would investigate which participants showed significant improvements and which did not.

To compute the RCI, we calculated 95% confidence intervals for change scores in the different test scores. We subsequently determined for each individual participant whether their individual change in a test score exceeded the 95% confidence interval or not. Individual-level changes beyond the 95% confidence interval indicate that the observed individual-level change can be regarded as a reliable individual-level change, as the probability that the change occurred by chance is below 5% (i.e., when using one-tailed testing).

Different methods to calculate the confidence interval have been proposed. First, the reliable change index can be calculated based on the standard error of prediction (*SE_pred_*) [11].

(3) $$SE_{pred}=SD_{t2}\cdot \sqrt{(1-r_{tt}^{2}})$$

*SE_pred_* is calculated by multiplying the *SD_t2_* of the variable with the root of one minus the squared T1-T2 correlation of the variable. The 95% confidence interval for this index can be calculated by multiplying the *SE_pred_* by +/− 1.96.

Second, Iverson [15] recommended using an updated version of the original formula based on the standard error of the difference (*SE_diff_*):

(4) $$SE_{diff}=\sqrt{{\left(SD_{t1}\cdot \sqrt{\left(1-r_{tt}\right)}\right)}^{2}+{\left(SD_{t2}\cdot \sqrt{\left(1-r_{tt}\right)}\right)}^{2}}$$

The *SE_diff_* is calculated based on both, the *SD_t1_*, the *SD_t2_*, and the T1-T2 correlation. A 95% confidence interval index can again be calculated by multiplying the *SE_diff_* by +/−1.96.

Both equations yield slightly different results. For example, if *SD_t1_* = 15, *SD_t2_* = 10 and the T1-T2 correlation is .80, the results are *SE_pred_* = 6 (CI = +/−11.76) and *SE_diff_* = 8.06 (CI = +/−15.80), respectively. In our study, we reported results based on both *SE_pred_ and SE_diff_*.

After computing the confidence interval, the individual-level change for each participant is evaluated by subtracting their T1 test score from their T2 test score and comparing the resulting difference score to the confidence interval. When computing the individual change score, it is recommended to take into account potential practice effects ([11], [41]). This can be achieved by using a true score estimate for the second test score [7].

(5) $$Y_{TRUE}=M_{t1}+r_{tt}\left(Y_{OBS}-M_{t1}\right)$$

*M_t1_* represents the T1 mean of the test. The formula controls the change from T1 to T2 by the retest reliability. *Y_OBS_* is the individual T2 score observed in the retest and *r_tt_* is the T1-T2 correlation. If the T2 test score of a participant was 120, the T1 sample mean was 100, and the T1-T1 correlation was *r_tt_* = .80, this results in *Y_true_* = 116. This value can then be used to determine the “true” difference between the T1 and T2 score.

*3. Differential continuity.* Differential continuity represents the rank-order consistency of a test score. This means that it indicates to what degree participants retain their rank order placement relative to the other participants from the first to the second testing. That is, it answers the question, to what extent participants who scored the highest at first testing still score the highest at second testing. Differential continuity is usually used to quantify the test-retest reliability of test scores. The test-retest reliability is of great relevance when deciding if a test score should be used for long-term individual level diagnostic decisions such as educational placement decisions. There is no specific standard for differential continuity, but it has been suggested that values of .80 or even .90 are needed [37]. With an SD of 15, a continuity value of .80 would lead to a margin of error (MoE) of 13.15 IQ points; a value of .90 would be associated with a MoE of 9.30 IQ points.

Differential continuity is evaluated by correlation coefficients. Pearson correlation was used in our analyses, dividing the covariance of T1 and T2 by the product of their standard deviations. There are no established standards for differential continuity. Watkins and Smith [37] recommended correlations greater than at least .80 for individual level diagnostic decisions.

(6) $$r_{tt}=\frac{Cov(t1,t2)}{SD_{t1}\cdot SD_{t2}}$$

*Adaption for intelligence research: When investigating the differential continuity of lower order intelligence test scores, one has to consider that these scores share a substantial amount of variance with the general intelligence score. Thus, the differential continuity of the lower order scores may be partially explained due to the stability of general intelligence [6]. To assess the stability of the variance unique to any specific certain lower order score, we computed correlation coefficients controlling for general intelligence at T1 (g1) using partial correlation.

(7) $$r_{tt|g}=\frac{r_{t1t2}-r_{t1g1}\cdot r_{t2g1}}{\sqrt{\left(1-{r_{t1g1}}^{2}\right)\left(1-{r_{t2g1}}^{2}\right)}}$$

The size of a correlation is limited by the variability of the measured score. In our sample, we only assessed students attending gifted classes, restricting the range of the intelligence scores and thereby limiting the size of the correlations [2] and potentially underestimating the differential continuity. When information on the variability of the scores of interest is available for the full, unrestricted population (for example from test manuals), the correlations can be corrected for range restriction [35,39].

(8) $$r_{12c}=\frac{S_{X}r_{t1t2}}{{\left(S_{X}^{2}r_{t1t2}^{2}+s_{t1}^{2}-s_{t1}^{2}r_{t1t2}^{2}\right)}^{\frac{1}{2}}}$$

In this formula, $r_{t1t2}$ is the observed correlation between the T1 and T2 score, $s_{t1}^{2}$ is the estimated standard deviation in T1, and $S_{X}$ is the standard deviation in the unrestricted population.

Lastly, the individual estimates of differential continuity of the different test scores can be used to estimate the reliability of the resulting ability profile. In contrast to the previously presented measures of differential continuity, the profile reliability considers all different test scores simultaneously (i.e., all correlations between the multiple test scores). Lienert and Raatz [21] provided the following formula:

(9) $${}_{prof}r_{tt}=\frac{\bar{r_{tt}}-\bar{r_{tT}}}{1-\bar{r_{tT}}}$$

$\bar{r_{tt}}$ is the mean differential continuity of all scores included in the profile and $\bar{r_{tT}}$is the average test score intercorrelation. The profile reliability increases with increasing differential continuity of the scores and with decreasing average intercorrelation. Profile reliabilities of .5 or larger are considered to be sufficient for profile interpretation [21].

*4. Ipsative continuity.* Ipsative continuity represents the stability of the *configuration of different scores* of the individual test taker over time. It therefore quantifies the stability of ability profiles. The analyses presented here answer the question to what extent the individual strengths and weaknesses remain the same across different times of measurement across all test takers. Ipsative continuity analyses inform the interpretation and use of individual cognitive strengths and weaknesses for individual level diagnostic decisions. If the identified strengths and weaknesses do not replicate significantly above chance level, one should not base interventions or placement decisions on this information.

In a first step, individual strengths and weaknesses have to be identified for each individual participant. To this end, we calculated the critical difference between the general intelligence score and the respective lower order scores for both test and retest. The critical difference indicates the limit that the lower order score deviation from the profile mean (general intelligence) has to surpass to be less than 5% likely to occur by chance. A formula to calculate the critical difference was provided by [20].

(10) $$D_{crit}=1.96\cdot SD_{Gx}\cdot \sqrt{2-(r_{g}+r_{Gx})}$$

*SD*_Gx_ represents the population standard deviation of the respective lower order score. *r_g_* represents the reliability of general intelligence (Cronbach's α) and *r_Gx_* represents the reliability of the respective lower order score. If the difference between a lower order score and general intelligence was positive and larger than *D_crit,_* it was classified as an individual strength. Similarly, if the difference was negative and larger than *D_crit,_* the score was classified as an individual weakness. For example, if the standard deviation is 15 and the reliability of the genergal intelligence score and the lower order score are .95 and .85, respectively, the difference between the two scores has to be greater than 1.96 * 15 * √(2 – (0.95 + 0.85)) = 13.15 to be considered statistically significant.

Once the individual strengths and weaknesses have been identified, the stability of these categorizations can be quantified. To this end, we used two different methods. Cohen's kappa [8] is a change-corrected metric for the estimation of agreement on nominal scale data. It is often used to assess the degree of agreement between different raters, but can also be used to assess the degree of agreement between categorisations at different times of measurement.

(11) $$\kappa =\frac{P_{o}-P_{e}}{1-P_{e}}$$

In the formula, P_o_ is the observed agreement among raters or times of measurment, and P_e_ is the probability of chance agreement. The resulting values can range from −1 and 1, with 0 indicating no systematic agreement, 1 indicating perfect agreement (i.e., a cogntive strength at T1 is still classified as a strength at T2), and −1 indicating perfect disagreement. Landis and Koch [19] provided guidance for the interpretation of kappa values, with .00–.20 indicating slight, .21–.40 fair, .41–.60 moderate, .61–.80 substantial, and .81–1.00 almost perfect agreement.

*Adaption for intelligence research: The interpretation of kappa is straightforward. However, the coefficient is affected by an uneven distribution of categories. If one category is much more prevalent than the others (in case of intelligence measurement most likely “no significant strength or weakness”), kappa results become unreasonably low [5]. We therefore used a second metric for the estimation of agreement on nominal scale data with Cramér's V, a transformation of χ^2^:

(12) $$V=\sqrt{\frac{\chi^{2}}{n(s-1)}}$$

*n* denotes the sample size and *s* the number of categories. The interpretation of this parameter is similar to that of other correlation coefficients [40]. Again, the value 0 indicates no systematic agreement, 1 indicates perfect agreement, and −1 indicates perfect disagreement.

*Adaption for intelligence research: It should be noted that it is also possible to investigate differential continuity continuously (e.g., [10]). However, in intelligence testing practice, individual strengths and weaknesses are usually assessed categorically. Thus, in most cases the stability of categorical judgements is tested when evaluating the long-term viability of profile interpretation in practice (e.g., [36]).

### When to use which temporal stability analysis method?

**Fig. 1:**
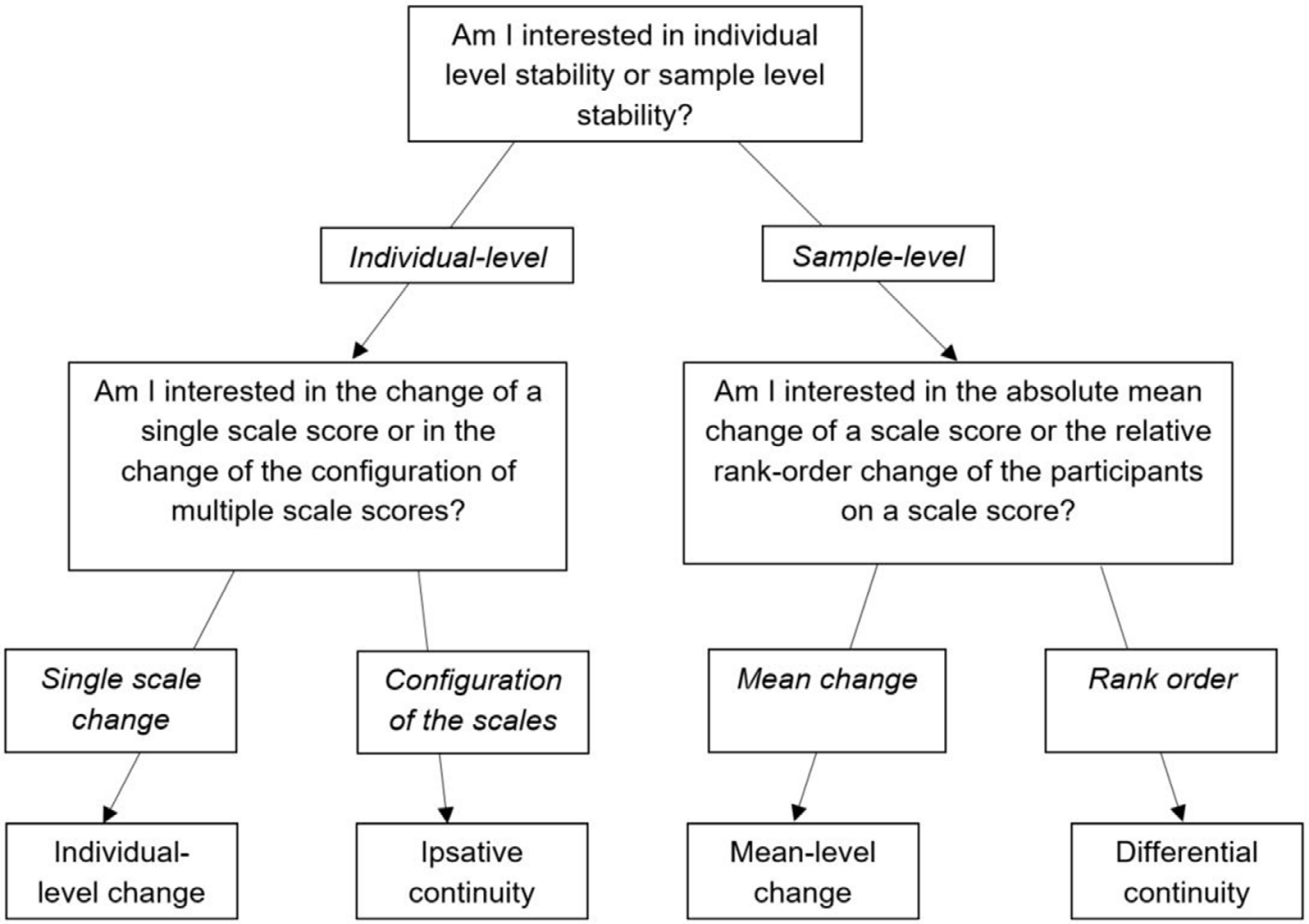
Decision guidance regarding which method is appropriate for which research question.

Temporal stability methods are complementary and not mutually exclusive [12,29]. Yet, for some research questions, it may be adequate to conduct one or two of the discussed methods in particular. Fig. 1 offers decision guidance for determining which method is appropriate for which research question. Two questions guide the decision process. The first question refers to whether one is interested in testing individual level stability or sample level stability. Depending on the answer, the second question either refers to (2a) whether one is interested in testing change in single scales or in the configurations of multiple scales within individuals or to (2b) whether one is interested in the absolute mean level change or the relative rank change of a scale score in a sample.

For example, if one's hypothesis is that a sample of older adults on average shows decreasing test scores over time, “sample level stability” and “mean change” are the answers to questions 1 and 2b respectively. These answers would guide one to the mean-level change method. If one's hypothesis is that some participants show decreasing scores on one scale over time while other individuals show no significant change, “individual level” and “single scale change” are the answers to questions 1 and 2a respectively. These answers would guide one to the individual-level change method.

## Real data application

### Sample

We assessed general intelligence and specific ability scores of 114 adolescents (mean age at T1 = 14.11; range = 12.67 to 15.67) from a gifted track of a German grammar school at two measurement points with a test-retest interval of six months. Testing took place in 2002 and 2003. Most students were male (71.1%). At both measurement points, 47 students were in 7^th^ grade (41.2%), 42 students were in 8^th^ grade (36.8%), and 25 students were in 9^th^ grade (21.9%). At T1, the average IQ of the students was 116.7 (*SD* = 9.97). We obtained written parental consent for all students. The sample was originally investigated in Breit et al. [4].

### Instrument

The BIS-HB [18] is a paper-and-pencil intelligence test designed to assess the intelligence structure of gifted students in particular. It can be administered both individually and in group settings. The test is based on the Berlin Intelligence Structure model (BIS; [17,34]), which is a faceted model comprised of an operation facet for Reasoning (R), Processing Speed (S), Creativity (C), and Memory (M) and a content facet for Figural (F), Numerical (N), and Verbal (V) ability. The two facets are conceptualized as orthogonal to each other, forming 12 cells defined by one operation and one content domain (e.g., reasoning – verbal, RV). The BIS-HB provides specific ability scores for each operation ability (R, S, C, & M) and domain ability (F, N, V) for ipsative profile analyses. Similar to other multidimensional intelligence tests like the WISC-V [38], the specific abilities scores are composite scores of the subtest scores associated with the respective specific ability.

## Results

The results are presented and interpreted in detail in Breit et al. [4]. The present result section illustrates how the different types of temporal stability can be presented. Further, it points out some differences and commonalities in the results attained from the different statistical methods used within the different types of temporal stability.

*1. Mean-level change.* In our sample, we found statistically significant increases of all scores from test to retest (Table 1). The mean increase across scores was 7.92 IQ points (*M_d_* *=* .61). The results show how t-tests and Cohen's d complement each other, indicating statistical significance and effect size, respectively.

**Table 1:** Means and Standard Deviations of BIS-HB Specific Ability Scores across a Test–Retest Interval of Six Months.

Ability Score	Test		Retest			
	*M*	*SD*	*M*	*SD*	Difference	*d*
Processing Speed	112.15	11.00	122.42	12.27	10.27	0.93
Memory	113.57	12.80	121.75	12.09	8.18	0.64
Creativity	109.15	12.66	116.34	13.04	7.19	0.57
Reasoning	118.04	9.96	123.63	9.64	5.60	0.56
Figural Ability	112.59	11.88	122.88	11.28	10.29	0.87
Numeric Ability	116.71	10.33	123.70	9.65	6.88	0.67
Verbal Ability	115.56	9.66	122.56	10.18	7.01	0.73
General Intelligence	116.72	9.97	125.48	9.61	8.78	0.88

*Note.* All differences *p* < .001

T-tests were calculated using SPSS (IBM [14]), indicating statistical significance for all score increases. We provide a real data illustration for the computation of Cohen's *d.* For Processing Speed, *M_t1_* *=* 112.15, *M_t2_* *=* 122.42, *SD_t1_* *=* 11.00, and *SD_t2_* *=* 12.27 (Table 1). Applying Eq. (2), this results in

$$d=\frac{122.42-112.15}{11.00}=\frac{10.27}{11.00}=.93$$

*2. Individual-level change.*Table 2 shows the percentage of participants who showed significant increases or decreases for the different test scores. The table is divided into classifications based on SE_pred_ and SE_diff_. Averaged across broad ability scores, 15.4% (SE_pred_) and 13.5% (SE_diff_) showed a reliable increase.

**Table 2:** Individual-Level Change of the BIS-HB Specific Ability Scores.

	Ability Score	% decrease	% same	% increase
SE_pred_	Processing Speed	0	76.3	23.7
	Memory	0	88.6	11.4
	Creativity	1.8	93.0	5.3
	Reasoning	0.9	83.3	15.8
	Figural Ability	0	78.9	21.1
	Numeric Ability	0.9	80.7	19.3
	Verbal Ability	0	88.6	11.4
	General Intelligence	0.9	68.4	30.7
SE_diff_	Processing Speed	0	76.3	23.7
	Memory	0	88.6	11.4
	Creativity	0.9	94.7	4.4
	Reasoning	0	88.6	11.4
	Figural Ability	0	80.7	19.3
	Numeric Ability	0.9	86.0	13.2
	Verbal Ability	0	88.6	11.4
	General Intelligence	0	70.2	29.8

*Note.* SE_pred_ *=* Standard Error of Prediction; SE_diff_ *=* Standard Error of Difference

We provide a real data illustration of the computation of the critical difference based on the Processing Speed data. First, we calculated the RCI based on *SE_pred_* (Eq. (3)), *SD_t1_* *=* 11.00 (Table 1), *SD_t2_* *=* 12.27 (Table 1), and *r_tt_* *=* .84 (Table 3), resulting in

$$SE_{pred}=12.27\cdot \sqrt{(1-.84^{2}})=6.65.$$

**Table 3:** Differential Continuity Coefficients and Differential Continuity Coefficients controlling for General Intelligence of the BIS-HB Specific Ability Scores.

Ability Score	*r_12_*	*r_12.g_*	*r_12c_*
Processing Speed	.84	.66	.91
Memory	.74	.59	.80
Creativity	.72	.55	.78
Reasoning	.84	.69	.93
Figural Ability	.77	.51	.85
Numeric Ability	.82	.53	.91
Verbal Ability	.81	.49	.91
Average *r*	.79	.57	.87
General Intelligence	.85	-	.93

*Note.* All coefficients *p* < .01. Correction for range restriction was based.

On the variability in the normative sample. *r_12_* *=* uncorrected autocorrelation.

*r_12.g_* *=* partial autocorrelation controlled for the general intelligence.

*r_12c_* *=* autocorrelation corrected for range restriction.

Multiplying *SE_pred_* *=* 7.02 by 1.96 yields the critical difference for reliable change of 13.05.

Second, we calculated the RCI based on *SE_diff_* (Eq. (4)). Applying the relevant Processing Speed values from Tables 1 and 3 results in

$$SE_{diff}=\sqrt{{\left(11.00\cdot \sqrt{\left(1-.84\right)}\right)}^{2}+{\left(12.27\cdot \sqrt{\left(1-.84\right)}\right)}^{2}}=6.59.$$

Multiplying *SE_diff_* by 1.96 yields the critical difference for reliable change of 12.92.

A participant of the present study had the following test values: Processing Speed at T1 *=* 108, Processing Speed at T2 (true score estimate) *=* 117.45, resulting in an increase of 9.45 points. This observed increase is smaller than 13.05 and 12.92, indicating that there was no reliable change for that participant based on both *SE_pred_* and *SE_diff_*.

*3. Differential continuity.*Table 3 presents the differential continuity coefficients for all BIS-HB scores. The uncorrected coefficients ranged from .72 to .84. The coefficients corrected for range restriction ranged from .78 to 93. When controlling for general intelligence, the stability of the lower order scores ranged from .49 to .69.

Lastly, the profile reliability was ${}_{prof}r_{tt}$= .53 when using uncorrected stability coefficients and ${}_{prof}r_{tt}$ = .71 when using the stability coefficients corrected for range restriction. Our sample showed range restriction in all scores compared to the BIS-HB standardization sample, resulting in substantially higher coefficients when correcting for this restriction. The results controlling for general intelligence imply that general intelligence accounts for a considerable portion of the stability of the lower order scores, but there is also significant stability of the respective unique variances.

We provide a real data illustration for the calculation of the correction for range restriction and the profile reliability for Processing Speed. The uncorrected autocorrelations and partial correlations controlling for general intelligence were computed using SPSS.

The uncorrected correlation between Processing Speed T1 and T2 was *r_t1t2_* *=* .84 (Table 3), the estimated standard deviation in T1 was $s_{t1}^{2}$ *=* 11, and the standard deviation in the unrestricted population was $S_{X}$ *=* 15.15. The correction for range restriction was calculated based on Eq. (8), resulting in

$$r_{12c}=\frac{15.15\cdot .84}{{\left(15.15^{2}\cdot .84^{2}+11^{2}-11^{2}\cdot .84^{2}\right)}^{\frac{1}{2}}}=.91$$

To calculate the profile reliability, we used Eq. (9), inserting the average intercorrelation of .55 and the average differential continuity of .79.

$${}_{prof}r_{tt}=\frac{.79-.55}{1-.55}=.53$$

Differential continuity values corrected for range restriction can be also used in this formula, yielding the profile reliability corrected for range restriction.

*4. Ipsative continuity.*Table 4 shows the agreement on strengths and weaknesses quantified by Cohen's kappa and Cramér's V. The median kappa value was *Mdn_κ_* *=* .34 (range .23–.58), indicating fair continuity. Median V was *Mdn*_V_ *=* .44 (range .22–.65), indicating moderate continuity. The higher continuity values indicated by Camér's V support the notion that Cohen's kappa may underestimate the stability when one category is overrepresented.

**Table 4:** Agreement on BIS-HB Ability Score-Based Strengths and Weaknesses Across a Test–Retest Interval of 6 Months, Indicating Ipsative Continuity.

Ability Score	*Κ*	V
Processing Speed	.25**	.22*
Memory	.29**	.33**
Creativity	.58***	.65***
Reasoning	.34***	.43***
Figural Ability	.34***	.45***
Numeric Ability	N.A.	N.A.
Verbal Ability	.23**	.30**
*Mdn κ*	.32	

*Note.* * *p* < .05, ** *p* < .01, *** p < .001. *κ* *=* Cohen's kappa.

V *=* Cramér's V. N.A. *=* not available because of 0 identified strengths and weaknesses at T1.

We demonstrate the calculation of the critical difference and apply the results to the data of a participant from our dataset. Eq. (10) was used to calculate *D_crit_* for Processing Speed, based on the population *SD* of Processing Speed (15) and the reliabilities of Processing Speed (.88) and general intelligence (.95) reported in the BIS-HB manual.

$$D_{crit}=1.96\cdot 15.00\cdot \sqrt{2-(.95+.88)}=12.12$$

A participant of the present study had the following test values: general intelligence at T1 *=* 117, Processing Speed at T1 *=* 108 (difference at T1 *=* −9 IQ points), general intelligence at T2 *=* 132, Processing Speed at T2 *=* 117 (difference at T2 *=* −15 IQ points). At T1, the difference is smaller than *D_crit_*, whereas at T2, the difference is greater than *D_crit_*, classifying Processing Speed as a cognitive weakness of the participant only at T2 and indicating disagreement between the two times of measurement. The degree of agreement between T1 and T2 classifications across all participants based on Cohen's kappa and Cramér's V was computed in SPSS.

## Discussion

We presented the statistical methods for the investigation of four different types of temporal stability of manifest intelligence test scores and illustrated their application with sample data; we further provide a decision guidance for choosing the most appropriate type of temporal stability analysis for a research question as well as the R code for the analysis protocol. We focused on the investigation of manifest test scores, which are frequently interpreted in intelligence testing practice. The methods presented allow evaluation of the usefulness of an intelligence test with regard to diagnostic decisions with long-term consequences. There are further research questions concerning the stability of intelligence, such as the temporal stability of the latent cognitive ability constructs (i.e., the g-factor). For these questions, modern statistical methods based on structural equation modeling or item response theory allow investigations of stability adjusted for measurement error. For example, latent change models and growth curve modeling can be used to investigate latent mean-level change (e.g., [32], [33]). Auto-correlative or auto-regressive structural equation modeling can be used to investigate latent differential continuity (e.g., [3,28]). Ipsative continuity can be tested by growth mixture modelling (e.g., [25], [26], [27]). Individual-level change can be investigated by observing the individual latent slope of a person from a growth curve model (e.g., [32]).

## Declaration of Competing Interest

The authors declare that they have no known competing financial interests or personal relationships that could have appeared to influence the work reported in this paper.

## Appendix Supplementary materials

Image, application 1
