# Supplementary material for: Temporal stability and change in manifest intelligence scores: Four complementary analytic approaches
Source: MethodsX. 2021 Dec 23;9:101613. doi: 10.1016/j.mex.2021.101613 (PMC8718888; doi:10.1016/j.mex.2021.101613)
Supplement: Supplementary file 1 [file mmc1.docx]

**Appendix**

### For the sake of clarity, we illustrate the methods using only the Processing Speed test scale ("s1" at T1 and "s2" at T2).

### The methods can analogously be applied to all other test scales by changing the variable names in the functions accordingly.

### install packages

install.packages("foreign")

install.packages("psych")

install.packages("ppcor")

install.packages("DescTools")

install.packages("rcompanion")

### load packages

library(foreign)

library(psych)

library(ppcor)

library(DescTools)

library(rcompanion)

### import dataset (in this case SPSS-dataset)

data <- read.spss("Data_Stability.sav", missing = -99, use.value.labels = F,to.data.frame = T)

##### mean-level change #####################################################################

# statistical significance: t-test

t.test(data$s1, data$s2)

# effect size: d

(mean(data$s2)-mean(data$s1))/sd(data$s1)

##### individual-level change ##################################################################

# Step 1a: reliable change index for Processing Speed based on SEpred

SEpred_s <- sd(data$s2)*sqrt(1-(cor(data$s1,data$s2)^2))

# Step 1b: reliable change index for Processing Speed based on SEdiff

SEdiff_s <- sqrt((sd(data$s1)*sqrt(1-cor(data$s1,data$s2)))^2 + (sd(data$s2)*sqrt(1-cor(data$s1,data$s2)))^2)

# Step 2a: confidence interval based on SEpred

CI_SEpred_s <- SEpred_s*1.96

# Step 2b: confidence interval based on SEdiff

CI_SEdiff_s <- SEdiff_s*1.96

# Step 3: new variable: difference in test scores between T1 and T2 in each participant based on a true score estimate of T2

data$diff_s <- (data$s1 + cor(data$s1,data$s2)*(data$s2-data$s1))- data$s1

# Step 4a: classify improvement, no change, and decline based on SEpred

data$change_pred_s <- ifelse(data$diff_s > CI_SEpred_s, "1", ifelse ((data$diff_s < CI_SEpred_s*(-1)), "-1", "0"))

# Step 4a: classify improvement, no change, and decline based on SEdiff

data$change_diff_s <- ifelse(data$diff_s > CI_SEdiff_s, "1", ifelse ((data$diff_s < CI_SEdiff_s*(-1)), "-1", "0"))

# Step 5a: percentages of improvement, no change, and decline based on SEpred

s_pred_table<-table(data$change_pred_s)

prop.table(s_pred_table)

# Step 5b: percentages of improvement, no change, and decline based on SEdiff

s_diff_table<-table(data$change_pred_s)

prop.table(s_diff_table)

##### differential continuity ##################################################################

# simple autocorrelation (r12)

cor.test(data$s1, data$s2)

# autocorrelation corrected for g [g1] (r12.g)

pcor.test(data$s1, data$s2, data$g1)

# autocorrelation corrected for range restriction (r12c), given SD in the unrestricted population = 15.15

rangeCorrection(cor(data$s1,data$s2), 15.15, sd(data$s1))

# profile reliability; given the average autocorrelation of .79 and average scale intercorrelation of .55

(.79-.55)/(1-.55)

##### ipsative continuity #####################################################################

# Step 1: Critical difference between g and Processing Speed (s) given the reliabilities of g (.95) and s (.88)

#and given the population SD of s (15)

D_crit_s <- 1.96*15*sqrt(2-(.95+.88))

# Step 2: Difference score s-g at T1 and T2

data$diff_s1_g1 <- data$s1-data$g1

data$diff_s2_g2 <- data$s2-data$g2

# Step 3: classify strength, weakness, or unremarkable at T1 and T2

data$swu_s1<- ifelse(data$diff_s1_g1 > D_crit_s, "1", ifelse ((data$diff_s1_g1 < D_crit_s*(-1)), "-1", "0"))

data$swu_s2<- ifelse(data$diff_s2_g2 > D_crit_s, "1", ifelse ((data$diff_s2_g2 < D_crit_s*(-1)), "-1", "0"))

# Step 4a: create crosstable, compute Cohen's kappa

xtab_s <- xtabs (~ data$swu_s1 + data$swu_s2)

# Step 4b: compute Cohen's kappa and Cramér's V

CohenKappa(xtab_s, conf.level = 0.95)

cramerV(xtab_s)
